# Patient Safety and the Question of Dignitary Harms

**DOI:** 10.1093/jmp/jhac035

**Published:** 2023-01-02

**Authors:** Polly Mitchell, Alan Cribb, Vikki Entwistle

**Affiliations:** King’s College, London, UK; King’s College, London, UK; University of Aberdeen, Aberdeen, UK

**Keywords:** dignitary harm, healthcare quality, iatrogenic harm, patient safety, respect

## Abstract

Patient safety is a central aspect of healthcare quality, focusing on preventable, iatrogenic harm. Harm, in this context, is typically assumed to mean physical injury to patients, often caused by technical error. However, some contributions to the patient safety literature have argued that disrespectful behavior towards patients can cause harm, even when it does not lead to physical injury. This paper investigates the nature of such dignitary harms and explores whether they should be included within the scope of patient safety as a field of practice. We argue that dignitary harms in health care are—at least sometimes—preventable, iatrogenic harms. While we caution against including dignitary harms within the scope of patient safety just because they are relevantly similar to other iatrogenic harms, we suggest that thinking about dignitary harms can help to elucidate the value of patient safety, and to illuminate the evolving relationship between safety and quality.

## I. INTRODUCTION

Patient safety is a central aspect of healthcare quality. Healthcare quality is an open-ended notion—often taken to encompass, for example, concerns about “patient-centered” and “equitable” provision, as well as clinical effectiveness and patient safety (Institute of Medicine, 2001, 6). Patient safety typically has a more determinate scope, focusing on the prevention of avoidable iatrogenic harm. *Iatrogenic harm* is harm which is not due to the underlying condition of the patient but results from medical actions or inactions. The definition of harm in this context, though rarely explicitly discussed, is largely assumed to be physical injury to patients. Some authors have argued that dignitary harms should also come within the scope of patient safety (Kuzel et al., 2004; Sokol-Hessner, Folcarelli, and Sands, 2015). Disrespectful, demeaning, and humiliating behavior towards patients, it is claimed, causes avoidable harm, even when it does not lead to physical injury.

This paper explores whether dignitary harms in health care are preventable, iatrogenic harms, and whether they should be included within the scope of patient safety as a field of practice. Such inclusion would have significant implications. It raises questions about whether the field is practically suited for this enlarged role and about healthcare improvement priorities. Safety is sometimes treated as a moral and practical priority within healthcare improvement because it is regarded as trying to secure the basic “floor” of good healthcare provision, in contrast to other more aspirational aspects of improvement that aim to raise the quality “ceiling” (Stevens, Matlow, and Laxer, 2005).^1^ Expanding the scope of patient safety could change this emphasis and might also represent a remodeling of the relationship between patient safety and healthcare quality more broadly conceived.

While we start to consider questions about the scope of patient safety in the final section, we chiefly concentrate on the central conceptual question of whether some dignitary harms qualify as preventable, iatrogenic harms, and are therefore suitable candidates for treatment as a patient safety concern. We argue that dignitary harms are—at least sometimes—best understood to be preventable, iatrogenic harms. Our analysis sheds light on the nature of harm, iatrogenesis, and preventability. We suggest that events cannot be understood to be iatrogenic without consideration of their proximity to central healthcare functions and their preventability. While we caution against including dignitary harms within the scope of patient safety just because they are relevantly similar to other iatrogenic harms, we suggest that thinking about dignitary harms helps to elucidate the value of patient safety, and to illuminate the evolving relationship between safety and quality.

## II. PATIENT SAFETY AND HARM

Harm is a crucial concept in health care. “Do no harm” has long been seen as a guiding principle, perhaps the primary principle, of medical ethics (Jonsen, 1978). Sometimes, this is taken to mean “do no *net* harm,” as benefitting a patient via medical treatment can involve harming them in some way (Sokol, 2013). However, there are harms that those delivering health care should seek to avoid in a more straightforward sense—notably those that are caused by medical error and the misuse of equipment or technologies. These non-instrumental harms, which cannot be warranted in terms of some further good, are the harms that patient safety takes as its subject (Kohn, Corrigan, and Donaldson, 1999).

“Patient safety” is a field of practice within health care, which has conventionally focused on medical error, the misuse of medical technologies, and resultant harms; it is also an attribute of healthcare systems—the safety of its patients—which the study and practice of patient safety seeks to underpin (Emanuel et al., 2008).^2^ Medical errors include “wrong side,” “wrong procedure,” or “wrong patient” surgery; leaving swabs or equipment inside patients after surgery; erroneous administration of medicines; and failure to maintain a sterile environment during intervention (Wilson and Sheikh, 2002; Seiden and Barach, 2006; Jung et al., 2019). These technical errors result, with varying probabilities and degrees of certainty, in harms to patients. Medical errors also include information errors such as the failure to take a complete medical history; inaccessibility or loss of patient notes; inadequate hand-over between staff; and insufficient explanation of disease management and symptom identification to patients (Wilson and Sheikh, 2002; Sheikh and Bates, 2014). Such errors may lead to avoidable ill health caused by misdiagnosis, late diagnosis, or missed opportunities for early intervention and mismanagement of disease.

Iatrogenic harms are often deemed to be preventable or avoidable. While there is not a single definition of “preventable,” there is wide agreement that a preventable harm has at least one modifiable cause, which is identifiable prior to any harm actually occurring, and that reasonable adaptation to a process prevents some future recurrence of the harm in question (Nabhan et al., 2012; Thomas, 2020). The practice and study of patient safety are, unsurprisingly, concerned with harms that can be prevented.

Characterizing an error as preventable does not entail that it was the result of a negligent action on the part of an identifiable individual or group of individuals. Patient safety, as a field of practice, has endeavored to move away from blaming individual practitioners for errors, recognizing that mistakes are often not due to individual incompetence or carelessness, but can result from systemic problems, including problems with organizational design, training and education, and equipment (Leape et al., 1995). The view of safety as an attribute of healthcare systems suggests errors can be reduced via design solutions, including not only the design of equipment and the physical environment, but also the design of processes and human systems (Emanuel et al., 2008). This “systems analysis” approach recognizes that well-meaning individuals, aiming to act in the best interests of patients, can nonetheless be led to make mistakes by poorly designed systems. It also supposes that accidents and mistakes are not tragically inevitable, but can be systematically analyzed to help prevent or minimize their recurrence.

While the definition—and the difficulty of defining—*preventable* harm is often discussed and disputed (Pronovost and Colantuoni, 2009; Nabhan et al., 2012; Papanicolas and Figueroa, 2019), the definition of *harm* per se is not typically deliberated in the patient safety literature. Most discussions of medical error do not set out a definition of harm. Many use “harm,” “adverse event” (or “adverse outcome”), and “injury” interchangeably, or define these concepts in terms of one another (Leape, 2002; Vincent and Coulter, 2002; Lamb et al., 2003; Brennan et al., 2005; Emanuel et al., 2008; Newman-Toker and Pronovost, 2009; Nabhan et al., 2012; Simpson, Aubin, and Fillatre, 2012; Papanicolas and Figueroa, 2019). Now each of these concepts has indeterminate scope—it is unclear what should be included within the category of “harm,” “injury,” or “adverse event,” and what should be excluded. Examples of preventable iatrogenic harm in the patient safety literature are almost always limited to major physical detriment—infections, bodily damage from unnecessary surgery, disease symptoms, and disease progression. Typically, the focus remains on harms caused by technical errors, including diagnostic errors (Newman-Toker and Pronovost, 2009; Singh and Graber, 2015). Detriments to psychological health are less often the focus of studies of iatrogenic harm, although it is relatively uncontroversial to suppose that major psychological harms should be included within the category of iatrogenic harm—medical error which induces or worsens the symptoms of a psychological illness is relevantly similar to medical error which worsens the symptoms of a physical illness. But there are other kinds of detriment that can be caused in the course of medical treatment, and which are therefore possible contenders for iatrogenic harms. Here, we focus on dignitary harms.

While there is much debate about its exact meaning and nature, there is widespread agreement that human beings have, or ought to have, something called dignity. Dignity is a distinctive form of moral worth, in virtue of which someone is worthy of respect and due particular kinds of treatment and consideration. Dignity is a central moral concept—often *the* central moral concept, since it is inter-defined with the concept of moral worth or moral status. It is invoked as the foundation for human rights (United Nations, 1948), as grounds for socio-relational equality (Anderson, 1999), and to explain why or to specify how we ought to act towards one another (Dworkin, 2000). Philosophers have variously emphasized the relationship between dignity and personhood and autonomy (Kant, 1996 [1785]); social status or position in a moral community (Darwall, 2006); capabilities (Nussbaum, 2006); and self-respect and authenticity (Dworkin, 2011). Dignity also plays a central role in contemporary medical ethics, although its meaning and value are contested (Macklin, 2003; President’s Council on Bioethics (U.S.), 2008; Cochrane, 2010). While some argue that the concept of dignity has no place in bioethics (Macklin, 2003), this is resisted, with others arguing that dignity plays an important role in forming and upholding social norms—a role that cannot be fulfilled by other related concepts such as respect for autonomy (Killmister, 2010; Hofmann, 2020). We do not seek to settle the meaning and nature of dignity here, merely to recognize that it is widely recognized as a foundational moral concept.

The nature of dignitary harm is, in part, a substantive question which this paper investigates; nonetheless, we set out a working definition. The concept of dignitary harm is relatively familiar in law and legal philosophy as a way of characterizing insults to a person’s dignity or autonomy, or injuries to their standing as a person (Brooks, 1999; Anderson, 2006). Dignitary harms are typically caused by disrespectful, humiliating, or dismissive conduct. Dignitary harms may be caused by words or conduct that treat someone as inferior on account of an assumed or actual group membership—racist, sexist, or transphobic behavior, for instance—or on account of personal characteristics—such as illiteracy or poverty. Examples of such conduct in healthcare contexts might include women being ignored or having information withheld from them during childbirth (Altman et al., 2019), or trans and non-binary folk being outed and not referred to by their preferred names and pronouns by administrative staff (Freeman and Stewart, 2018). Dignitary harms may also be caused by conduct that insufficiently considers the interests or dignity of a person regardless of their group membership. Consider, for example, a care assistant leaving a patient to sit or lie in their own excrement for an extended period of time; a hospital porter transporting a patient through public spaces in a state of undress; or a consultant loudly discussing sensitive, personal information about a patient in the presence of strangers. While dignitary harms are sometimes called “emotional harms” (Sokol-Hessner, Folcarelli, and Sands, 2015), they are probably best understood not to be identical to emotional harms, because not all emotional harms need be status-related or dignity-affecting, and someone may not be aware of all potential harms to her dignity. Dignitary harms, then, capture those non-physical harms that involve treating someone as if they were a moral or social inferior (Barclay, 2018). The importance of dignity in bioethics and moral philosophy more broadly warrants taking assaults on dignity—of the kind characterized by dignitary harms—seriously. Even those who deny the significance of dignity per se might nonetheless recognize that treating others with disrespect or as inferior is morally problematic, perhaps because it undermines other core values or principles of biomedical ethics.

A number of contributors to the patient safety literature suggest that disrespectful behavior is relevant to patient safety because it creates conditions in which medical errors are more likely to occur, and so causes preventable iatrogenic harms, understood in the orthodox sense (Entwistle, 2008; Leape et al., 2012; Martinez et al,. 2017; Sokol-Hessner et al., 2018). This might be because disrespectful attitudes and behavior inhibit teamwork between staff members; discourage patients from engaging in full and frank conversations with their doctors, or lead them to disengage from medical care altogether; or because such attitudes mean that clinicians do not listen to or believe their patients when they raise concerns or otherwise offer information salient to their care. This can lead to the kinds of medical errors and resultant physical harms discussed above. This suggests that disrespect is *instrumentally* relevant to traditional patient safety concerns. There is some evidence to support these claims, although much appears to be anecdotal (Blanchard and Lurie, 2004; Leape et al., 2012).

Now several authors have further suggested that the non-physical impact of disrespectful attitudes and behavior on patients can *itself* constitute a harm, which should be considered to be within the scope of patient safety (Kuzel et al., 2004; Entwistle, 2008; Rees, 2012; Sokol-Hessner, Folcarelli, and Sands, 2015; Sokol-Hessner et al., 2018). Sokol-Hessner, Folcarelli, and Sands (2015) suggest that disrespectful attitudes and behaviors that result in emotional harms should be given the same attention that patient safety has given to physical harms. They recommend that these harms should be recorded, categorized, and have their severity assessed, as is currently standard with respect to physical harms. In one study, primary-care patients reported psychological harms far more frequently than physical or economic harms (Kuzel et al., 2004). “Psychological harms” included emotions such as anger and frustration, feelings of belittlement and a sense of violation, diminished trust in clinicians, and anxiety about health. Many of the feelings and states included under this category are negative mental and emotional states that do not necessarily amount to clinical mental health issues. At least some of these “psychological harms” are caused by disrespectful conduct and involve insults to dignity and status. The authors argue that the public views patient safety through a lens different from the medical community, one which prioritizes emotional and psychological harms over physical ones.

This literature suggests that disrespect is not just instrumentally relevant to traditional patient safety concerns, but that disrespectful behavior *is itself* a patient safety concern. This implies that patient safety as a field of practice has systematically overlooked a whole category of harm—dignitary harms. The success of this challenge depends on dignitary harms being sufficiently similar to the harms conventionally discussed within patient safety—that is, actually being preventable, iatrogenic harms. In the following two sections, we consider whether dignitary “harms” are, first, in fact harms and, second, iatrogenic.^3^ Subsequently, in the final section of the paper, we return to the implications of these discussions for the fields of patient safety and quality improvement.

### Are Dignitary “Harms” Harms?

Broadly speaking, a harm is something that is bad for us. But, usually “harm” is reserved for a subset of things that are bad for us, distinguished perhaps by their severity—being worse than mere hurts, say—by their ramifications—suggesting the necessity of remedial or preventative action—and potentially by their genesis—describing purposeful or negligent, rather than purely accidental, injury. In this section, we explore the definition of harm to assess whether what we have called dignitary “harms” are in fact best understood to be harms.^4^

With a few exceptions, discussion of harm in bioethics does not explore the definition and scope of harm. Discussion of harm-benefit and risk-benefit calculations rarely includes mention of what should and should not be included in these calculations (Gillon, 1994; Jackson, 2006; Beauchamp, 2007; Seedhouse, 2008; Sokol, 2013; Veatch, 2016). Those contributors that do identify the need to define the nature and scope of harm do not go on to provide such an analysis. Beauchamp and Childress (2013), for instance, brush over the definition of harm very quickly. Their discussion of harm focuses on physical harm and while they acknowledge that there are also psychological harms, they do not discuss the nature of these or attempt to provide a more exhaustive account of the scope of harm. Seedhouse (2008) acknowledges that what counts as a harm, and which harms are worse than others, is up for debate, so a maxim like “do no harm” cannot be invoked without a degree of interpretation. Now this assumes that we already know broadly what kinds of things should be included in the category of relevant harms, such that disagreement occurs at the margins, in deciding where the boundaries should be drawn in particular cases. The challenge of dignitary harms represents a more significant disagreement—that is, whether a whole category of harms has been overlooked by patient safety.

The idea that the invocation to “do no harm” might involve healthcare professionals being careful in their practice—consistent with the practice of patient safety—is occasionally articulated (Jonsen, 1978; Beauchamp and Childress, 2013). Jonsen (1978) argues that it is unethical for a doctor to act carelessly in diagnosis and therapy, because harm may result. Beauchamp and Childress (2013) also recognize that “non-maleficence” involves healthcare professionals taking due care, though what counts as “reasonable prudence” is context dependent. Neither discussion, however, explores what kind of carelessness—carelessness with respect to what end—is relevant for ascertaining whether someone is behaving unethically. Carelessness about keeping equipment sterile is presumably relevant, while carelessness with respect to polishing shoes is probably not. But what about carelessness with respect to use of patients’ preferred name and personal pronouns? Or carelessness in leaving cubical curtains open when a comatose patient is being washed or dressed? Can such behaviors generate iatrogenic harms? Or are they at most wrongful behaviors moral agents should avoid, but not ones that generate harms, or have particular relevance to assessment of medical practice? In this section, we look to other areas of philosophy that have generated more explicit discussions of the nature of harm than bioethics, most notably legal philosophy (Harman, 1981; Feinberg, 1984; Linklater, 2006; Dan-Cohen, 2009; Simpson, 2013).

An influential account of harm, defended by Feinberg in *Harm to Others* (1984), defines harms as wrongful setbacks to interests. Feinberg distinguishes between two senses of harm. In the first, “non-normative” sense, to harm is to thwart, set back, or defeat someone’s interests. In the second, “normative” sense, to harm is to wrong someone, or to treat that person unjustly; Feinberg suggests that such harms are often also harmful in the first sense.^5^ As Feinberg points out, not all setbacks to interests are wrongs—often someone’s actions invade our interests excusably or justifiably, such as when we consent to their so doing. An interest, here, is understood to be something in which someone has a stake, that is, where they stand to gain or lose depending on its nature or condition. Interests comprise *ultimate interests*—a person’s significant projects, causes, and goals—and the more minimal *welfare interests* required to sustain these, which include: continuing to stay alive; health and normal bodily functioning; absence of absorbing pain; minimal intellectual acuity; emotional stability; a tolerable social and physical environment; and the absence of groundless anxieties and resentments.^6^

Feinberg distinguishes between “harms,” “hurts,” and “offences.” Hurts are minor physical pains (tinges, aches, throbs), “painful” mental states (keen disappointment, remorse, grief), and unpleasant physical sensations (itches, dizziness, weakness, stiffness). Offences are non-painful but unpleasant mental states. They include disgust, irritation, frustration, shame, embarrassment, anger, and fear. Hurts and offences are not, for Feinberg, harms, because we do not have an interest in not being hurt or offended as such.^7^ He argues that a hurt is sufficiently serious to qualify as a harm if and only if it is either a symptom of a prior harm of another order or else it is in itself the cause of a consequential harm of another order. So, for example, incessant frustration, fear, or irritation may, over time, compromise emotional stability or lead to major psychological breakdown, which would amount to a setback to interests and so a harm. In such cases, the psychological breakdown would be harmful, rather than the frustration or irritation per se.^8^

If harms are best understood as setbacks to interests, are dignitary harms plausibly harms? Dignitary harms can be causal factors in physical harms which occur in healthcare contexts. Demeaning behavior toward patients may discourage them from accessing the healthcare system, and from learning about their own condition and its management. However, dignitary harms appear, on this account, to *cause* harms but not to *be* harms. Dignitary harms seem mainly to comprise what Feinberg calls offences, and perhaps sometimes what he calls painful mental states. While they are in some sense bad, they perhaps do not affect interests enough to amount to harms. This would suggest that intervening to prevent them from occurring in healthcare contexts would be overly constraining and interfering, and so unjustified. One way of challenging this assessment might be to assert that we have an *interest* in equal moral status or in dignity, which is violated by dignitary harms.^9^ Behavior that casts someone as morally and socially unequal does not merely hurt feelings or cause that person to feel shame, anger, frustration, but rather—or also—it fails to recognize that person’s moral status, fails to recognize her as a person, in ways that are prohibitive of her flourishing. If dignity can be thought of as a kind of welfare interest, then on Feinberg’s own terms dignitary harms could be counted as harms.

However, this argument depends on the demeaning behavior in question actually succeeding in undermining equal moral status or dignity. Simpson (2013) argues that we ought to maintain a distinction between speech acts that express the view that someone is inferior—on the basis of group membership, for instance—and those that actually succeed in diminishing their status within a community. Claims that particular speech acts “assault,” “infringe,” or “violate” someone’s dignity are, he suggests, ambiguous between these two interpretations. Simpson argues that even if we recognize that facts about someone’s moral status are dependent on facts about how they are seen and treated by other people in their moral community, this need not imply that someone’s being treated as a second-class citizen means that they have in fact been made a second-class citizen. Such reasoning ascribes too much impact to the behavior of certain individuals. This suggests that even if it is possible that *some* dignitary harms are genuine harms, in reality, most are unlikely to be so.

According to Feinberg’s second, “normative” sense of harm, wrongful conduct can be harmful. If dignitary harms are *wrongful*, then they may be harmful even if they do not set back any interests. The nature and boundaries of wrong action are highly contested and whether dignitary harms are wrongs seems potentially even more intractable than whether dignitary harms are harms. Now allowing (some) dignitary harms the status of harms solely on the basis of their being wrongs seems dis-analogous to the way in which physical harms are taken to constitute the typical focus of patient safety. A physical harm, in patient safety, is normally taken to refer to some kind of non-trivial injury or damage caused by healthcare failures, rather than a wrong-doing per se, even when these two things co-occur. The practice of patient safety is also interested in “near misses”—errors which contingently did not result in any harm, but which easily could have. These, perhaps, could be conceptualized as wrongs that are non-harmful in the setbacks to interests’ sense, insofar as they put patients at unjustified risk of harm. Now there is reason to resist the idea that such errors are necessarily wrongful. Indeed, the field of patient safety has done much to challenge the assumption that medical errors and any resulting harms are wrong. Sometimes medical errors result from negligent conduct on the part of individuals or teams. But, patient safety’s systemic approach emphasizes that although events that are characterized as medical errors are outcomes of systems and processes that are in some sense *worse* than identifiable and achievable counterfactual scenarios, this need not mean that patients’ rights are violated, or that anyone can meaningfully be said to have either wronged or been wronged. There is thus, we think, reason to avoid characterizing the harmfulness of dignitary harms merely in terms of their wrongfulness. The purpose of patient safety, and its interest in avoiding harm, is different from that of the criminal law—Feinberg’s subject—and it is perhaps not surprising that harm should be characterized differently in these divergent contexts. This is not to say that, when a medical error or dignitary harm *is* wrongful, its wrongfulness is not another reason to think that it should be mitigated and prevented. But it does suggest that its wrongfulness might not be relevant to its inclusion within the scope of patient safety.

We return, then, to consider whether dignitary harms might be understood to be setbacks to interests. The distinction between harms and mere hurts or offences seems, for Feinberg, to be at least partly a matter of degree. While we do not have an interest in avoiding minor pains, injuries, and distress as such, we do have an interest in avoiding major pains, injuries, and distress, insofar as these incapacitate us with respect to our welfare interests, and so constitute setbacks to our ultimate interests. There is, then, a question as to whether and when dignitary harms are debilitating to this extent—especially if they often fail to cause their target to become socially inferior. Of course, not all instances in which someone is treated as inferior result in them being *entirely* unable to pursue and fulfill their personal projects, and so sustain their ultimate interests. Thus, not all setbacks to health, emotional stability, economic sufficiency, and so on, entirely preclude us from pursuing our ultimate interests, so it is not clear that this should prevent treatment as a moral equal from being a welfare interest. An infection that someone acquires during medical treatment necessitating a 30-day hospital stay is harmful, even if it does not prevent her from pursuing her passions in the longer term.

This draws attention to an ambiguity in the distinction between harms and mere hurts and offences in Feinberg’s account. For something to count as an impediment to someone’s ultimate interests, it has to obstruct them in some sense. But, determining whether something has impeded her interests, and so whether it is a harm rather than an offence, is not straightforward. A requirement that her ultimate interests should be precluded entirely by some event or conduct in order for it to be treated as harmful sets the bar far too high. This would rule out all sorts of characteristic harms. Now allowing that something can harm her by merely delaying her pursuit of her ultimate interests for a few minutes or hours, or making it a little more difficult for her to pursue them, seems to set the bar too low. This might make very minor detriments—a failed attempt to take a blood sample or a 20-minute unexpected wait—into harms.

This highlights a further difference between the criminal law-context in which Feinberg is working and the patient safety context. The systemic approach of patient safety does not just involve identifying harms that cross a certain threshold of harmfulness, but thinking more broadly about the comparative advantageousness or harmfulness of different states of affairs. That is, patient safety conceives of an event as harmful insofar as it makes someone worse off relative to a specified baseline or relative to other courses of action, rather than insofar as it has some specifiable characteristics. How to specify the baseline for comparison is a somewhat open question—it could be defined descriptively, in terms of current practice or usual practice, or it could be defined proscriptively, in terms of good or ideal or best possible practice (Wilkinson, 2003). Importantly, thinking about harm in the patient safety context is likely to be more concerned with the comparative harmfulness of different actual and potential courses of action, rather than trying to discern a threshold for what counts as a harm or not. In the patient safety context, then, an action’s capacity to make things worse for someone is more central to understanding its harmfulness than its surpassing a given threshold in this respect.

A second ambiguity in the relationship between offences and harms concerns the point at which cumulative offences become harms. Feinberg sometimes suggests that cumulative offences can *cause* harms, and sometimes that they can *qualify as* harms or *be* harmful. The distinction between these may in practice be quite difficult to discern. Consider someone who is subject to recurrent disrespectful treatment in the course of their medical care—for example, a woman with a severe physical disability whose clinical team speak only to her partner about her condition, despite her lacking any communicative impairment or mental incapacity. Such behavior might make her extremely distressed, severely damage her sense of agency and self-worth, and impact on her relationship with her partner. Has this behavior *caused* or *constituted* a setback to her interests? We might want to say that it causes her to become emotionally unstable, which would imply that it *causes*—but not necessarily constitutes—a setback to her interests. If this conduct impairs her relationship with her partner, then it might be an impediment to one of her central ultimate interests. Now we might also want to say that the disrespectful behavior *constitutes*—rather than causes—an intolerable social environment, which could be understood as a setback to her interests. In the first case, and possibly the second too, the disrespectful behavior might not be strictly harmful, although it causes harm, whereas in the last case the disrespectful behavior can itself be understood to be harmful. There is, then, potential ambiguity in the way that offences become harms, once sufficiently severe or numerous.

Central to Feinberg’s distinction between hurts and harms is the claim that we do not have an interest in not being hurt or offended *as such*. Feinberg appears to mean by this that we do not have an interest in not being subject to particular hurts or offences, unless those hurts and offences cause or are caused by a harm. But this suggests that we cannot determine whether a given hurt or offence is something that we *do* have an interest in avoiding without understanding its context. This would likely include understanding its causal relation to other actions, hurts, and outcomes; its cultural, ethical, and linguistic significance in the setting in which it transpired; its consequences for the individual in question; and how it fits into their personal history. If a particular hurt or offence *is* part of a harmful pattern of behavior, there seems to be good reason to treat it as harmful, even if it is not harmful “as such,” that is, even if it would not have been harmful, had it occurred in context without such pattern of behavior.

Dignitary harms are notable for being cumulative. A single instance of humiliation, fear, or degradation might not be harmful, but the cumulation of years of dismissive criticism and discriminatory treatment of particular individuals and groups of individuals by people in positions of power can make particular disrespectful acts harmful or liable to harm in a way that they would not be in absence of this history. The same conduct or words might be deeply disrespectful when targeted at one person, but barely disrespectful at all when directed at another. So, thinking about the harmfulness of particular dignitary harms involves thinking about their place in the life of the person whose status or identity is in question. Disrespectful conduct toward women, ethnic, and racial minorities, queer and trans folk, people with disabilities, and members of other disadvantaged and marginalized groups has the potential to be especially injurious and may be more liable to cause harm than similar words and behavior aimed at more privileged people. This is especially likely to be true if the disrespectful attitudes explicitly or implicitly refer to marginalized characteristics. Even microaggressions have the potential to be harmful in contexts of repeated, long-term disrespect, and insubordination (Sue et al., 2007; Freeman and Stewart, 2018). Furthermore, the same conduct or words might be disrespectful, or *more* disrespectful, because of facts about the speaker or the relationship between the speaker and the recipient. Demeaning words or public humiliation from a friend, a mentor, or someone who is supposed to be caring for us may be more likely to damage our interests than the same words or behavior from someone in whose views we do not put much stock. This suggests that fairly substantial contextual knowledge is needed to understand the impact of disrespectful conduct, and so its propensity to undermine dignity.

The upshot of all this is that there is not a neat solution to the question of whether dignitary harms are best understood as harms. Even a framework like Feinberg’s, which prima facie appears to clearly distinguish harms and mere offences, leaves a substantial grey area between harms and non-harms. However, if treatment as a moral subordinate is liable to incapacitate someone with respect to their significant projects, causes, and goals, then there seems to be reason to understand that treatment as harmful. We think that it would be difficult to defend the view that *all* disrespectful behavior is harmful, but it would also be difficult to defend the view that *none* is harmful. There is good reason to think that dignitary harms are at least the kind of thing that warrant consideration as harms in the context of patient safety: they can make things worse—sometimes much worse—for those who suffer them. The discussion in this section has raised a number of features of dignitary harms worth noting. Identifying the harmfulness of dignitary harms may require greater historical, cultural, and contextual knowledge than is needed to identify the harmfulness of physical harms. This potentially makes it quite difficult to recognize dignitary harms. Moreover, while determining the exact point at which someone’s interests have been set back is indistinct even with respect to physical harms, the sense in which dignitary harms set back her interests may be even more ambiguous. For if the definitions of the interests in question—a tolerable social environment or emotional stability, for instance—are more contested than those that are typically disrupted by physical harm—such as continuing to stay alive or normal bodily functioning—then it will be more difficult to secure agreement on the characterization of a given instance of disrespectful behavior as harmful. However, such epistemic limitations need not imply that dignitary harms cannot be harms, nor that they should not be treated as such. Indeed, similar limitations can also characterize the identification of physical harms.

### Are Dignitary Harms Iatrogenic?

Even if dignitary harms are plausibly *harms*, in order to determine whether dignitary harms are a potential concern for patient safety, we must also determine whether they are *iatrogenic*. Patient safety is specifically concerned with harms resulting from *health care*, and if it turns out that dignitary harms are not best understood in this way, then they may fall outside of its remit. In this section, we first consider reasons for thinking that dignitary harms are non-iatrogenic. We then discuss the relationship between iatrogenesis and preventability, and argue that dignitary harms that are precipitated or legitimated by healthcare (or proximate) professionals should be understood to be iatrogenic.

If it were stipulated that only harms caused by clinicians could be classed as iatrogenic, then dignitary harms caused by receptionists, hospital porters, managers, technicians, or other non-clinical staff would not be iatrogenic harms. However, as noted above, patient safety is concerned with the correct functioning of systems, including the activities of a variety of people and of kinds of “hardware” and “software.” Consider some examples:

- *Medical records:* a patient suffers iatrogenic harm due to misdiagnosis; her X-ray images were erroneously filed by a clerk in the medical records office, so her consultant was not given the evidence needed to make a correct diagnosis.
- *IT bug:* a wrong site surgery is performed due to an IT bug which resulted in incorrect information being provided to the surgical team.
- *Laundry:* an outbreak of antibiotic-resistant infections occurs in a ward because the company contracted to provide laundry services used inappropriate washing procedures.

It is plausible to think of these as instances of iatrogenic harms. Health care depends not just on clinicians, but on a network of clinical and non-clinical individuals, often in multiple institutions. If non-dignitary iatrogenic harms can be caused by non-clinical actors, then dignitary harms cannot be ruled out as iatrogenic simply because they are caused by non-clinical actors.

One concern with this argumentative strategy is that it potentially inflates the definition of iatrogenesis beyond its normal or serviceable usage. Very many systems and factors are necessary for the effective running of the healthcare system: the tax system, transport infrastructure, schools and universities, and the farming and food production industries, for instance, but also the water cycle, the absence of epidemic infectious disease, and the supply of fuel and other resources. Does this suggest that these systems and factors are part of the healthcare system? Or that harms caused by failures of the healthcare system that result from failures of these supporting systems should be understood to be iatrogenic? Rather than either allowing the definition of health care to be grossly expanded, or insisting on an artificial boundary around a set of institutions that are designated healthcare institutions, we suggest that the boundaries of health care can be indistinct. Some supporting systems are much more proximate and specific to the delivery of health care than others: medical education and training, the pharmaceutical industry, hospital cleaning services, and healthcare administration, for instance. Harms resulting from failures of or errors in these systems are more plausibly deemed iatrogenic harms than harms resulting from more remote and non-healthcare specific systems, such as for electricity generation. Crucial to determining whether dignitary harms are iatrogenic harms, then, will be ascertaining whether their causes are relevantly proximate or specific to core healthcare institutions.

Underlying the suggestion that dignitary harms might not be iatrogenic harms is a suspicion that the conduct and harms involved do not appear to be connected to the distinctive practices or purposes of health care. Consider a hospital receptionist who deadnames and misgenders a trans patient in a public space^10^; or a midwife who makes a pointed comment about absent fathers to a pregnant black woman whose partner is unable to attend an antenatal appointment. In such cases, it might seem that insofar as such disrespectful conduct is harmful, its harmfulness is not due to a technical healthcare error, but a more general social or moral error. A teacher, shopkeeper, or colleague could have been responsible for comparable disrespectful conduct, and we could thus understand it to be a result of harmful human interaction rather than harmful healthcare practice. If dignitary harms occur in health care, *at the same time* as health care is being provided, but not strictly *as a result of* health care, then it might be misleading to call them iatrogenic harms. Dignitary harms in health care are perhaps not sufficiently specific to the good functioning of healthcare institutions to be considered iatrogenic.

It should be clear from the discussion thus far that health care should be taken, for the purpose of defining iatrogenesis, to at least include aspects of clinical practice such as history taking, diagnosis, and follow-up care, and not just treatment-directed aspects of care, such as the prescription of therapies and surgical intervention. Now this need not imply that everything that happens within a healthcare space when a patient is receiving health care is part of health care. Nor need it imply that all *harms* that happen within such spaces and periods of time are iatrogenic harms. Reflection on some “edge-cases” of iatrogenic harm may help to tease out its scope a little further. Consider the following incidents:

iv. *Mosquito*: a hospice patient is bitten by a mosquito and develops malaria.
v. *Step*: a patient trips over a step in a GP surgery and breaks her wrist.
vi. *Fight*: a fight breaks out between two patients in an emergency waiting room, one of whom cuts open his head on a chair.

At first glance, it might seem like a stretch to characterize any of these as iatrogenic harms. Each occurs within a healthcare setting, but the cause of the harm is not a product of health care. But adding some contextual detail puts pressure on these intuitions:

vii. Mosquito*: The hospice has neither installed window mesh nor provided permethrin-treated bed nets, despite being situated in an area with high malaria risk.
viii. Step*: It is the fifth time this year that someone has been injured in the same way. The step has not been marked with hazard tape despite having been reported to the practice manager more than once.
ix. Fight*: The hospital has recently refurbished their emergency department, but did not employ evidence-based design principles which have been shown to reduce aggression and violence in similar contexts.

In the expanded examples, the healthcare institution has some degree of knowledge, or the opportunity for knowledge, about a risk of harm, and in each case has not acted on it.

The contextual details by no means conclusively demonstrate iatrogenesis in these cases, but if the expanded descriptions *more plausibly* describe iatrogenics than the contracted versions, then they bring to light some factors which might be relevant to establishing whether a harm should be treated as iatrogenic or not. First, they highlight that healthcare institutions can be more or less responsive to known risks, both quite general environmental risks, as in mosquito*, and more local risks, as in step*. Of course, the type and amount of evidence about the probability, severity, and consequences of a risk can vary, so there might be stronger evidence for the causal link between inaction and harm, and more potential casualties, in mosquito* than in fight*, for instance. Sometimes a risk might be so probable or so severe that it is clearly careless or negligent for it not to have been recognized or acted on. One factor indicating iatrogenesis, then, might be that the harms in question occur when a healthcare institution knows, or can reasonably be expected to know, that they are liable to occur and yet have not taken any precautionary action. Second, the cost and difficulty of taking action to reduce the risk can vary. So, for example, the cost of action in step* may be far lower than the cost of action in mosquito* and fight*. But it is also worth considering that in fight* the cost of preventative action while the area was being refurbished would be a lot less than otherwise. A second factor indicating iatrogenesis, then, might be the ability of healthcare and healthcare-proximate institutions to take preventative action.

In order to determine whether dignitary harms are best understood to be iatrogenic, two issues therefore need to be addressed. First, whether the social attitudes and behaviors that are liable to cause dignitary harm are sufficiently proximate or specific to health care to be considered relevant to iatrogenesis. And second, whether dignitary harms are realistically identifiable and preventable by healthcare institutions. We consider each in turn.

One way of assessing whether dignitary harms that occur during the course of health care are best considered iatrogenic harms is to consider the functions of a healthcare system. The functions of a healthcare system shape the practices that are more and less proximate to health care. If the absence of dignitary harms is central to a healthcare system’s functioning, or if dignitary harms are irreconcilable with its functions, this might be reason to consider them to be iatrogenic. Paradigmatic iatrogenic harms are, plausibly, iatrogenic because it is part of the function of doctors and medical care to produce health benefit, or prevent deterioration of health, by treating, curing, and managing disease. The fact that paradigmatic iatrogenic harms arise from failures to fulfill the central functions of medicine also seems to connect to the moral and practical priority that attaches to the field of patient safety. Conduct undermining these central functions—by failing to prevent deterioration, or by introducing new threats to health, for instance—causes harm that is *iatrogenic.* On the same basis it can be argued, for example, that a company providing laundry services for a hospital which causes an outbreak of antibiotic-resistant infections by following incorrect bedlinen washing procedures causes iatrogenic harm because it undermines a function of health care. Exactly what this function is might come under various descriptions: so it might be part of the function of a hospital to maintain an environment that does not systematically undermine recovery, or it might specifically be one of the functions of a hospital to prevent antibiotic-resistant infections, as far as is reasonably possible.

Is it plausible to suppose that it is part of the function of healthcare institutions to treat patients respectfully, or to avoid humiliating and demeaning behavior? If the aim of health care is simply to improve biomedical function, then it might look, prima facie, as if the answer to this question is “no.” However, disrespect and humiliation threaten forms of communication and interpersonal relationships that are important for improving and sustaining biomedical function. Even medicine that is not explicitly “co-productive” involves patients deciding when to approach healthcare services to ask for help, cooperating with questioning, examination, and testing, following treatment plans, attending follow-up appointments, recognizing symptomatic improvement and deterioration, and so on (Batalden et al., 2016). Biomedical success is thus premised in central ways on the engagement, understanding, and cooperation of patients with respect to their health, the healthcare system, and healthcare professionals. This is particularly apparent with respect to long-term conditions, the management of which chiefly takes place outside of healthcare institutions (Entwistle, Cribb, and Owens, 2018). To the extent that effective health care depends on patients’ contributions, disrespectful treatment that undermines a person’s agency, self-respect, or confidence can threaten their motivation and ability to engage with healthcare institutions.

But the aims of health care are not limited to improvement in biomedical function. It is not controversial to suppose that clinical effectiveness is only one aspect of good health care. The U.S. Institute of Medicine’s multi-dimensional definition of healthcare quality specifies that good health care is—among other things—patient-centered, which means that it is “respectful of and responsive to individual patient preferences, needs, and values” and equitable, which means “care that does not vary in quality because of personal characteristics such as gender, ethnicity, geographic location, and socioeconomic status” (Institute of Medicine, 2001, 6). The U.K. Care Quality Commission definition specifies that health care must be caring, where this means that staff treat patients with “compassion, kindness, dignity and respect” (Care Quality Commission, 2018). And, the World Health Organization says that health care must be people-centered, which means that it “takes into account the preferences and aspirations of individual service users and the culture of their community” (World Health Organization, 2019a). These broader definitions of healthcare quality—to which clinical success contributes, but which it does not exhaust—see health care as functioning in service of a wide set of personal and social goods. All three see respectful, dignified treatment as an end of good health care in itself. A more expansive conceptualization of the purpose of health care—which is largely consistent with these multidimensional accounts—might see health care to be in service of the broader aim of enabling people to live and die well (Morgan et al., 2017). Sometimes this will involve the treatment and cure of disease, sometimes it involves helping people to live and die well with their conditions. From this perspective, dignitary harms can be understood to sometimes undermine the fulfillment of the purpose of health care, insofar as they interfere with patients’ well-being more generally (Entwistle, Cribb, and Owens, 2018).

Because disrespectful, undignified conduct is widely taken to be a violation of some of the core functions of health care, there is prima facie reason to see dignitary harms in health care as iatrogenic. If dignitary harms are not preventable by healthcare institutions, either because they are too costly to prevent, or because there is limited evidence as to which actions will prevent them, then there may be reason to ultimately exclude them from the category of iatrogenic harms. It is outside the scope of this paper to assess the evidence for the effectiveness of interventions to prevent dignitary harms, so we assume that it is at least possible that effective interventions to mitigate disrespectful conduct can be developed. Disrespectful behavior, particularly where someone’s membership in a marginalized or disadvantaged group is grounds for their treatment as inferior, is a society-wide problem, fostered by historical power structures and marginalization. The task of preventing disrespectful behavior might simply seem like too great a task for healthcare institutions to resolve. It is all very well to demand that healthcare staff must treat patients with dignity and respect but, in cultural contexts where the perceived inferiority of particular social groups is deeply embedded in citizens’ attitudes, language, and expectations, this is far easier said than done. Fixing such a complex knot of problems might seem like too large a burden to impose on healthcare institutions. Healthcare institutions seem more a stage on which dignitary harms are played out than their ultimate source.

However, healthcare institutions should not be allowed to evade responsibility for dignitary harms so easily. Some dignitary harms are specific to healthcare contexts, or much more liable to occur within them. In such cases, responsibility cannot easily be palmed off onto other institutions or actors. Discrimination on the basis of race, gender, sexuality, disability, and class can and does occur everywhere. But the nature of healthcare environments enables some kinds of discrimination or gives them a distinctive character. Certain dignitary harms in health care are made possible because of the sensitive and intimate nature of situations into which patients can be placed. For example, the possibility of dignitary harms which involves lack of sensitivity around public nakedness arises in healthcare contexts because medical care sometimes involves patients undressing, or wearing gowns which allow easy access to parts of their body. Similar situations are unlikely to occur in most other public institutions. Furthermore, dignitary harms relating to the stigmatization of diseases and conditions—including sexually transmitted diseases, mental health issues, and physical or intellectual disability—might be distinctively harmful in healthcare contexts because these are expected to be environments in which these conditions are taken seriously, managed, and treated. Dignitary harms relating to childbirth occur mostly in healthcare contexts because the vast majority of births across the world are assisted by healthcare attendants (World Health Organization, 2019b).^11^ In contexts such as these, there is good reason to put the onus on healthcare organizations to address these dignitary harms.

Moreover, action taken to address disrespectful and demeaning behavior has to start somewhere, and healthcare institutions are conceivably well-placed to take a lead. On the one hand, they tend to employ very large numbers of people, compared to other industries. In the UK, healthcare employment accounts for as much as 10% of all employment in some areas (Reed et al., 2019). Initiatives in health care to target discriminatory behavior from staff could thus reach a large number of citizens as potential perpetrators. Moreover, almost all citizens engage with healthcare institutions at several points during their lives, so initiatives that successfully reduce discriminatory behavior by healthcare staff could impact a large number of citizens as potential beneficiaries. As “anchor institutions” that are embedded in their communities, large healthcare institutions such as hospitals have the potential to use their employment and procurement power to support local economies (Reed et al., 2019). Analogously, there might also be potential for such institutions to use their assets and influence to support local communities through the respectful treatment of patients and visitors. Other social institutions might also fruitfully take on a similar role, but healthcare services potentially reach a broader segment of the community than schools and universities, local and national government, emergency services, and similar organizations.

## III. DIGNITARY HARMS AND THE FIELD OF PATIENT SAFETY

Should dignitary harms fall within the scope of patient safety? Our arguments suggest that some dignitary harms can be understood to be preventable, iatrogenic harms and so are suitable candidates for inclusion. But this only takes us so far—our progress with relevant conceptual questions has not resolved their practical implications. In this concluding section, we begin to consider these practical concerns. Deciding whether dignitary harms should fall within the scope of patient safety is more than a conceptual question. Patient safety is a “real world” field of practice within the broader field of quality improvement. Incorporating dignitary harms into the field of patient safety raises a range of questions that we can only touch on here. For example, would such incorporation undermine the field’s organizing rationale, and how far is it feasible to reshape the field without damage? Would it obscure the clarity of focus and motivating purpose of work on patient safety?

Paradigmatic examples of patient safety failures are clinical failures that result in physical damage with potentially significant long-term implications for patients. Both the implications of such damage and the fact that they represent the opposite of what health care is supposed to achieve, give addressing them a moral and practical priority. They make patient safety “matter” to a degree that does not always apply to every aspect of healthcare quality. The arguments we have rehearsed suggest that some dignitary harms can sit securely within such a sharply defined focus. For example, health services that are specifically directed toward supporting people with intellectual or physical disabilities would fail in their central function if they generate identity-damaging forms of stigmatization. It does not seem especially controversial to argue that addressing such harms should be an important patient safety concern. Other kinds of dignitary harms map less perfectly onto the paradigmatic cases and may thereby blur the conventional emphasis somewhat, along with the distinction between “threshold” safety concerns and aspirational quality considerations. The practical implications of expanding the field of patient safety thus partly depend on the set of dignitary harms in question, and how adaptable the field can be expected to be. In our remaining remarks, we offer some cautions about hastily expanding the field of patient safety, but also some reflections on the longer-term growth and evolution of the field which suggest a more positive conclusion.

Recognizing patient safety as a practical field of activity involves, among other things, recognizing that it has associated roles, policies, and methodologies—albeit ones with fuzzy boundaries. Practical fields are path-dependent; they are shaped by historical decisions and circumstances, which limit the decisions that can be made about them in the future, and they cannot be enlarged or re-shaped quickly or without consideration of the costs and benefits of so doing. In other words, patient safety should be treated as a historically determinate, solution-oriented, and pragmatic field, rather than a domain whose scope is fixed by the definition of preventable, iatrogenic harm. This pragmatic characterization of the study and practice of patient safety highlights a gap between what would improve patient safety, as an attribute of systems, *in theory* and what would improve it *in practice*. Changing the field of patient safety to be more conceptually coherent does not necessarily translate to improvements in the safety of patients. For this reason, we would suggest that dignitary harms should only be included under the patient safety banner if and when this enables the prevention of dignitary harms and does not hinder the prevention of other iatrogenic harms. Our arguments suggest that dignitary harms should be afforded similar attention to those in the traditional domain of patient safety. However, this need not necessarily imply that the field of patient safety should seek to rapidly or radically change and expand its remit—unless there is good reason to think that doing so will strengthen patient safety efforts. Caution seems to be a sensible short-term response.

However, looking further ahead, things are more complicated. Patient safety is an evolving field and its evolution is blurring boundaries. It is unsurprising that several people have argued that patient safety should attend to dignitary harms; these arguments correspond to other ways in which the field of patient safety has been expanding its boundaries. Seeing harms as system products, instead of focusing on the mistakes of individual clinicians, brings an indefinitely large range of actors and processes into the purview of patient safety as potential causes of harm or modes of harm prevention. A related, but more recent and far-reaching, revision of the scope of patient safety has been the advocacy of the “Safety-II” paradigm (Hollnagel, Wears, and Braithwaite, 2015). Safety-II draws on “complex systems” theories to critique traditional patient safety approaches (“Safety-I”) as too simplistic—over-concentrating on things that go wrong and explaining these as analogous to “mechanical breakdowns” where simple causal lines can be drawn between faulty components or connections and safety problems. By contrast, advocates of Safety-II stress the necessity of ongoing adaptation and performance variability within dynamic systems. They argue that students of safety should focus at least as much on understanding how and why things “go right” as well as “go wrong.” This arguably shifts the center of gravity of patient safety as a field of practice so that its domain of interest begins to converge with the broader study of healthcare quality. The growing prominence of worries about over-diagnosis and over-treatment also transcends the field of patient safety and reinforces this blurring of boundaries (see Glasziou et al., 2013; Levinson, Born, and Wolfson, 2018). This rising agenda highlights how an emphasis on mistakes—poor clinical practice or system breakdowns—may miss important kinds of harm that arguably result from systems and clinicians working essentially as expected. In order to capture the nature of the harms produced by expected functioning, we have to be ready to open up debates about the purposes of health services, which include consideration of dignitary harms.

There is no question that respect is an important healthcare quality consideration: it is highlighted in all influential accounts of quality. Nor is there disagreement about whether what we have called “dignitary harms” have instrumental relevance for underpinning patient safety. What remains contestable is whether the prevention of dignitary harm (or some dignitary harms) should figure as an independent aim of patient safety, and not merely as contributors to physical harms. It is possible that such contestation may not be long-lasting, given the evolution of the patient safety field and the possibility that some of the distinctions we have considered may have less relevance over time. In light of a Safety-II approach, it is difficult to maintain, for instance, that dignitary concerns are highly distinctive because of their “cultural” (as opposed to the material) constitution. Furthermore, although the distinction between the instrumental and non-instrumental value of dignity within safety is clear-cut in the abstract, it may be more ambiguous in practice.

Dignitary harms in healthcare contexts do have distinctive characteristics. There is likely to be greater disagreement around the identification of dignitary harms compared to physical and clinical psychological harms. Identifying dignitary harms involves not just a causal analysis of the events, but also attention to what was *intended* and how events were *interpreted*. Such considerations may also be relevant to identifying communication and information errors, and their associated harms. The interpretative aspects of identifying dignitary harms add a layer of contextual complexity to orthodox patient safety approaches. Understanding whether and why behavior is disrespectful warrants linguistic and cultural analysis, and sometimes knowledge about patients’ identity, values, and preferences. Where the identification and mitigation of dignitary harms involves different kinds of analysis and intervention, including much more detailed cultural and contextual enquiry, from current patient safety efforts, there may be reason to keep the former separate. But the differences are arguably ones of degree only, and good scholarship about traditional safety subjects—such as medical complications arising from invasive tubing and lines—now focuses on the “sociocultural” as well as the “technical” dimensions of healthcare quality (Kriznik, Lamé, and Dixon-Woods, 2019).

Established approaches to combining sociocultural and technical lenses in the analysis of patient safety can be helpful for thinking about the prevention of dignitary harms. Dignitary harms can present a problem of many hands, such that it can be difficult to attribute responsibility to a single individual (Dixon-Woods and Pronovost, 2016). Take, for example, a patient being exposed because their bed-curtain is left open while they are being washed or dressed. The causal factors involved might include a broken curtain rail; high seasonal demand for beds; staffing gaps in the hospital maintenance department; reduced departmental and hospital income; national austerity measures; and so on. Or, consider a trans patient who is repeatedly deadnamed by healthcare staff. Causal factors may include the design of patient information systems, which makes it difficult to record preferred names and pronouns or fails to display preferred names; disjointed IT systems and incomplete data sharing across primary and secondary care; lack of a local or general convention of asking people how they prefer to be identified; and widespread transphobic attitudes. In such cases, the carelessness of staff is only one of a wide range of causal factors, and not necessarily the primary one. Mitigating dignitary harms requires similar sociocultural and technical system-level analysis to that used to assess the causes and means of prevention of other iatrogenic harms.

A strengthened focus on disrespectful behavior is one of the next challenges for patient safety, regardless of whether dignitary harms are iatrogenic harms in their own right. Unprofessional or disrespectful behavior is rarely challenged, even when it is liable to lead to iatrogenic harms (Leape et al., 2012; Martinez et al., 2017). However, in practice, it is likely to be difficult to distinguish those instances of disrespectful conduct harmful in their own right, and those merely liable to cause or contribute to other harms. For it is not just that particularly egregious instances of disrespectful behavior—or certain kinds of disrespectful behavior—cause poor communication, distrust, and disengagement from healthcare services, and then lead to iatrogenic harms. Rather, ordinary forms of disrespectful behavior have these effects, too. This suggests that trying to keep dignitary harms that are not instrumental to other iatrogenic harms out of patient safety, while keeping dignitary harms that *are* instrumental to other iatrogenic harms in, might be something of a fool’s errand.

So, there seems to be good reason for the remit of patient safety to expand, at least over time, to include dignitary harms, both as instrumental and non-instrumental harms. Because of their distinctive qualities, there is also reason to attend carefully to the manner in which dignitary harms are addressed in patient safety efforts. The more conventional tools of patient safety—briefings, checklists, protocols, warning systems—cannot necessarily be assumed to be useful in the mitigation of dignitary harms, or not without significant remodeling. The inherently interactional and relational aspects of disrespect, and the high level of cultural specificity that attaches to dignitary harm, need to be taken into account in order to identify and think about preventing such conduct and its consequences. Seeing dignitary harm as a branch of patient safety enables other iatrogenic harms to be cast in a new light. Furthermore, these new and more diffuse ways of thinking about patient safety seem to correspond with the way the field is already evolving. Exploring the harmfulness and iatrogenesis of disrespectful behavior shows that patient safety need not be just a matter of ensuring that clearly defined institutions or actors avoid or achieve clearly defined ends. Instead, understanding and identifying avoidable, iatrogenic harms involves thinking about the nature and function of health care, its practicable limits, and the way that it depends on and interacts with other institutions.

## Footnotes

^1^
The perceived importance of patient safety is sometimes expressed by its being treated as an independent object of study alongside healthcare quality, rather than merely a dimension of quality. The title of one of the leading journals in this field—*BMJ Quality & Safety*—is a case in point.

^2^
These two conceptions of patient safety are, of course, not distinct from one another. Patient safety, as a field of practice, aims to support and realize the safety of patients through research and professional practice. However, the two may come apart: the study and practice of patient safety may not always succeed in improving the safety of patients.

^3^
Preventability, we argue, is a necessary condition for iatrogenesis.

^4^
We continue to use the phrase “dignitary harms”—despite calling into question whether they are in fact best conceptualized as harms—in order to maintain the link to discussion of insults to a person’s dignity elsewhere in the legal and philosophical literature.

^5^
We include scare quotes here to register our uncertainty that these two senses of harm are in fact best characterized as, respectively, non-normative and normative.

^6^
Feinberg develops this bipartite definition of harm as part of a broader project exploring when it is legitimate for the state to punish and coerce people. For Feinberg, harms that are both wrongful *and* setbacks to interests demand some kind of intervention to prohibit or penalize them, this dual requirement setting a relatively high bar and preventing too much state intervention.

^7^
Feinberg acknowledges that the distinction between offences and hurts is to a certain extent arbitrary, but he upholds it because offences are a category of particular interest. The key distinction is between harms and non-harms.

^8^
This example highlights the fact that hurts may have a cumulative significance, a consideration to which we return shortly.

^9^
While we do not set out to prove this, we take dignity to be at least a conceivable candidate for an interest on Feinberg’s account: that is, we plausibly have stake in our dignity, and we stand to gain or lose depending on its condition.

^10^
A deadname is the birthname of someone who has changed it. To deadname someone is to refer to them by their birthname. These concepts are most commonly used in the context of trans and non-binary people who have changed their names.

^11^
There is a further question about whether, and under what circumstances, the presence of a healthcare professional makes something into a healthcare context. It would stretch the concept of iatrogenesis to suggest that all dignitary harms occurring in the presence of a healthcare professional are thereby iatrogenic, but their ability to mitigate or prevent the harm in question could constitute one indicator of iatrogenesis.
